# Can energy saving and emission reduction policies promote green transformation of industrial enterprises——The Case of China

**DOI:** 10.1371/journal.pone.0301891

**Published:** 2024-05-06

**Authors:** Chunyan Li, Deqi Wang, Rui Hu, Fei Zhang, Mingna Li

**Affiliations:** 1 School of Urban Economics and Public Administration, Capital University of Economics and Business, Beijing, China; 2 School of Statistics, Shanxi University of Finance and Economics, Shanxi, China; 3 School of Statistics, Beijing Normal University, Beijing, China; Chengdu University of Information Technology, CHINA

## Abstract

In the context of the continued advancement of the green economy transition, the proactive pursuit of carbon emissions reduction and the early attainment of carbon neutrality goals have emerged as essential components in promoting high-quality economic development. Not only does it contribute to the creation of a community of human destiny, but it is also vital to the realization of sustainable development for human civilization. A dynamic evolutionary game model, which encompasses the interactions among government, enterprises, and the public, was constructed to examine the inherent impact mechanisms of the behavior of three players on the development of a green economy under the context of energy saving and emission reduction subsidies. The results showed that the incentive and punishment mechanisms served as effective tools for harmonizing the interests of system members. Within the mechanisms, the public demonstrated a higher sensitivity to rewards, while enterprises exhibited greater responsiveness to fines. Consequently, the government could influence the behavior of enterprises by incentivizing the public to serve as a third-party inquiry and oversight body. Simultaneously, the government could encourage enterprises to expedite green technology innovation by employing a combination of incentive and punishment mechanisms.

## 1. Introduction

With the rapid development of industrialization, the excessive emission of carbon dioxide and other greenhouse gases has led to an increase in the earth’s temperature, causing great damage to both human society and the ecological environment [1]. Energy and environmental problems have become increasingly prominent and have become a primary constraint on the rapid development of the economy and society [2]. According to the United Nations "Climate Change 2021: The Physical Science Basis" report, the increase in carbon emissions has led to accelerated warming of the atmosphere, oceans, land, and the frequent occurrence of extreme weather events, such as heat waves, heavy precipitation, droughts, and typhoons, which have posed serious challenges and crises to sustainable socio-economic development. How to seek a balance between realizing economic development and considering the carrying capacity of environmental resources has become an urgent strategic issue that needs to be addressed urgently. China, the world’s largest carbon dioxide emitter, needs to make more efforts to reduce carbon dioxide and greenhouse gas emissions as well as transform its economy and energy system [3]. How to quickly and effectively realize greenhouse gas emission reduction and timely complete the carbon neutral target that China has promised is a major realistic problem in China’s current development process.

To achieve the goal of carbon neutrality, it is necessary for the government to reduce carbon emissions through energy saving and emission reduction policies, such as taxation or subsidies for green innovation [4]. Energy saving and emission reduction policies refers to the constrained and incentives policies tools adopted by the government with the policies goals of reducing greenhouse gas (GHG) emissions. Constrained policy tools are mainly used to increase the cost of carbon emissions of enterprises through administrative intervention [5, 6] and legal regulation [7, 8] to force high-carbon enterprises to use more clean energy and take the initiative to reduce emissions [9]; Incentive policies tools primarily involve the government combining the costs and benefits of enterprises through subsidies and taxes [10–12]. In Welfare Economics, Pegu mentioned that due to economic externalities and other reasons, the maximization of social welfare cannot be achieved. Hence, it is necessary for governments to address these issues through subsidies for incentives or taxation. This will not only mobilize the enterprises to actively reduce emissions but also effectively solve the contradiction between carbon emission reduction and economic development. In practice, since energy saving and emissions reduction incentives are supported by government financial expenditures, the design of appropriate subsidy measures becomes particularly crucial [13].

Currently, China is in the latter stages of industrialization, characterized by high investments, high energy consumption, high carbon emissions, and low efficiency in its industrial production sector. About 65% of China’s total energy consumption is derived from the industrial sector, making it the country’s largest energy consumption [14]. Taking coal energy as an example, China’s total energy consumption amounted to 5.41 billion tons of standard coal in 2022, and coal consumption accounted for 56.2% of total energy consumption. Within this, the proportion of coal consumption in industrial production exceeded 90% of the national total. Therefore, promoting carbon emission reduction in the high-energy-consuming industrial sector plays a crucial role in accelerating the green transformation of development, sounding the industrial system, and achieving the sustainable development of the socio-economic and ecological environment, which is not only a vital link in realizing green development but also serves as an important means of developing a low-carbon economy. At the same time, the Chinese government has given strong support for energy saving and emission reduction policies in the transition to a low-carbon and green economy. For example, China’s State Council issued the Comprehensive Work Program for Energy saving and Emission Reduction in the 14th Five-Year Plan to vigorously promote energy saving and emission reduction, accelerate the establishment and improvement of a green, low-carbon and recycling development of the economic system, and promote the comprehensive green transformation of economic and social development to realize the goal of carbon peak and carbon neutral. In particular, the Law of the People’s Republic of China on Energy saving, as amended in 2018, with its fifth chapter explicitly proposes to support energy saving and emission reduction through financial subsidies and tax incentives. The launch of the above laws and administrative regulations has provided a legal and regulatory basis for subsidizing energy saving and emission reduction. Therefore, the energy saving and emission reduction subsidy policies, as an important macroeconomic regulatory tool for the Chinese government to realize the low-carbon transformation, is of great practical significance to study the intrinsic influence mechanism of China’s green economy development.

## 2. Literature review

At present, research on energy saving and emission reduction policies mainly focuses on the following two aspects: 1) Assessing the effects before and after the initiation of energy saving and emissions reduction policies [15–18]; 2) Conducting simulation analyses of the implementation effects of energy saving and emission reduction policies under various scenarios [19–22].

From the perspective of comparative studies of energy saving and emission reduction policies conducted before and after their launching, most scholars choose to study the effectiveness of China’s carbon trading market on green economic growth, urban pollution control, and corporate green innovation following the initiation of China’s carbon trading market. Acemoglu et al. (2016) believe that taxes and subsidies play an important role in incentivizing the production and innovation of clean technologies [16]. Nauleau et al. contend that differential subsidies can achieve social efficiency optimization [15]. Zhang (2022) argues that government subsidies can enable cleaner production enterprises to benefit far more from subsidies than heavily polluting enterprises, thereby incentivizing enterprises to innovate in a green way and to reduce the intensity of pollution emissions in the production process [23]. Zheng et al. (2023) regarded the pilot "comprehensive demonstration city of energy saving and emission reduction fiscal policies" as a quasi-natural experiment, and after a difference-in-differences model with multiple time periods was constructed, they found that the energy saving and emission reduction fiscal policies significantly enhanced the quality and quantity of green technological innovation in the demonstration city [24]. Zhang (2023) based on the asymptotic double difference method to analyze the impact of green fiscal policies on enterprises’ green innovation and found that the comprehensive demonstration city pilot significantly promoted enterprises’ green innovation, and this promotion role presents a dynamic and continuous feature that increases year by year [25].

From the study of a simulation of energy efficiency and emission reduction policies, the main methods applied include the difference-in-differences model [19]、 evolutionary game model [21]、 system dynamics model [26]、 dynamic stochastic general equilibrium (DSGE) model [27] and so on. Nie et al. (2016) constructed a model of the output subsidies of energy efficiency under a duopoly situation, arguing that a certain amount of subsidies can reduce the carbon emission intensity of enterprises [28]. Li (2020) constructed a system dynamics model of energy consumption and pollutant emissions of industrial enterprises in Shanxi Province to visually examine the energy saving and emission reduction effects of various environmental regulation policies, providing a realistic basis for industrial environmental regulation policy innovation in resource-based regions [26]. Zhang (2020) empirically investigated the impact of energy saving and emission reduction policies on urban eco-efficiency using the difference-in-differences model, finding that energy saving and emission reduction policies significantly improve urban eco-efficiency [19]. Wu (2017) established a three-sector dynamic stochastic general equilibrium (DSGE) model to study the dynamic impact of energy saving and emission reduction subsidies, manufacturers’ energy saving and emission reduction efforts, and other measures on China’s macroeconomy [27]. Chen (2021) constructed an evolutionary game model between manufacturing firms and the government. It was found that the firms eventually engaged in green and low-carbon innovation after the government was compensated, rewarded, or penalized for the innovative behavior of manufacturing enterprises in the form of transfer payments and tax credits [21]. Zhang (2022) used a two-party game model to analyze the behavioral strategies of the government and non-state-owned enterprises (NSOEs) in the process of realizing the "dual-carbon" goals.

Currently, in the process of energy saving and emission reduction policies research, most of the studies focus on the analysis of the game behavior between the government and enterprises. Some studies also focus on establishing a three-party evolutionary game among enterprises, government, and verification agencies to examine the interactions and constraint mechanisms among these players [29, 30]. Few studies have included the public as players in the game model and considered a comprehensive balance of interests in collaborative governance among governing subjects. However, the public, as an important part of the development process of the socio-economic system, has been included in the game model in other areas of research, such as water resource governance [31], land governance [32], ecological governance [33], urban governance [34], and so forth. Therefore, this paper introduces the private sector, emphasizes the role of non-governmental organizations and the private sector in the governance of carbon emission reduction, and constructs a tripartite game model of "government-enterprise-public". At the same time, this paper advocates for multiple governance and multilevel governance to explore the behavioral choices of different game subjects, and provide decision support for the government to incentivize and guide enterprises to achieve optimal economic benefits and energy saving and emission reduction benefits through the development of low-carbon economy. This paper makes a minor contribution to the research: Firstly, it introduces the public participation based on traditional government-enterprise cooperation mode, and a three-sector collaborative governance game model of "government-enterprise-public" is constructed, which provides a basic framework for the analysis of inter-subject interaction; Secondly, combined with the specific development of China’s energy saving and emission reduction policies, systematically analyze the evolution and stabilization strategy combinations of the incentive and punishment mechanisms by central and local governments in different scenarios. It deeply analyzed the intrinsic mechanism of carbon emission reduction inherent in the synergistic governance of collaborative governance among multiple subjects in carbon emission reduction; Finally, using Matlab R2016b software for simulation analysis, constructed the selection path of multiple games among players, analyzed the change process and influencing factors of the behavior of all players in the production process of low-carbon economy. Based on the analysis results, put forward measures and suggestions for the government to improve the carbon reduction and emission reduction co-governance mechanism.

## 3. Methodology

### 3.1 The evolutionary game model

**Fig 1:**
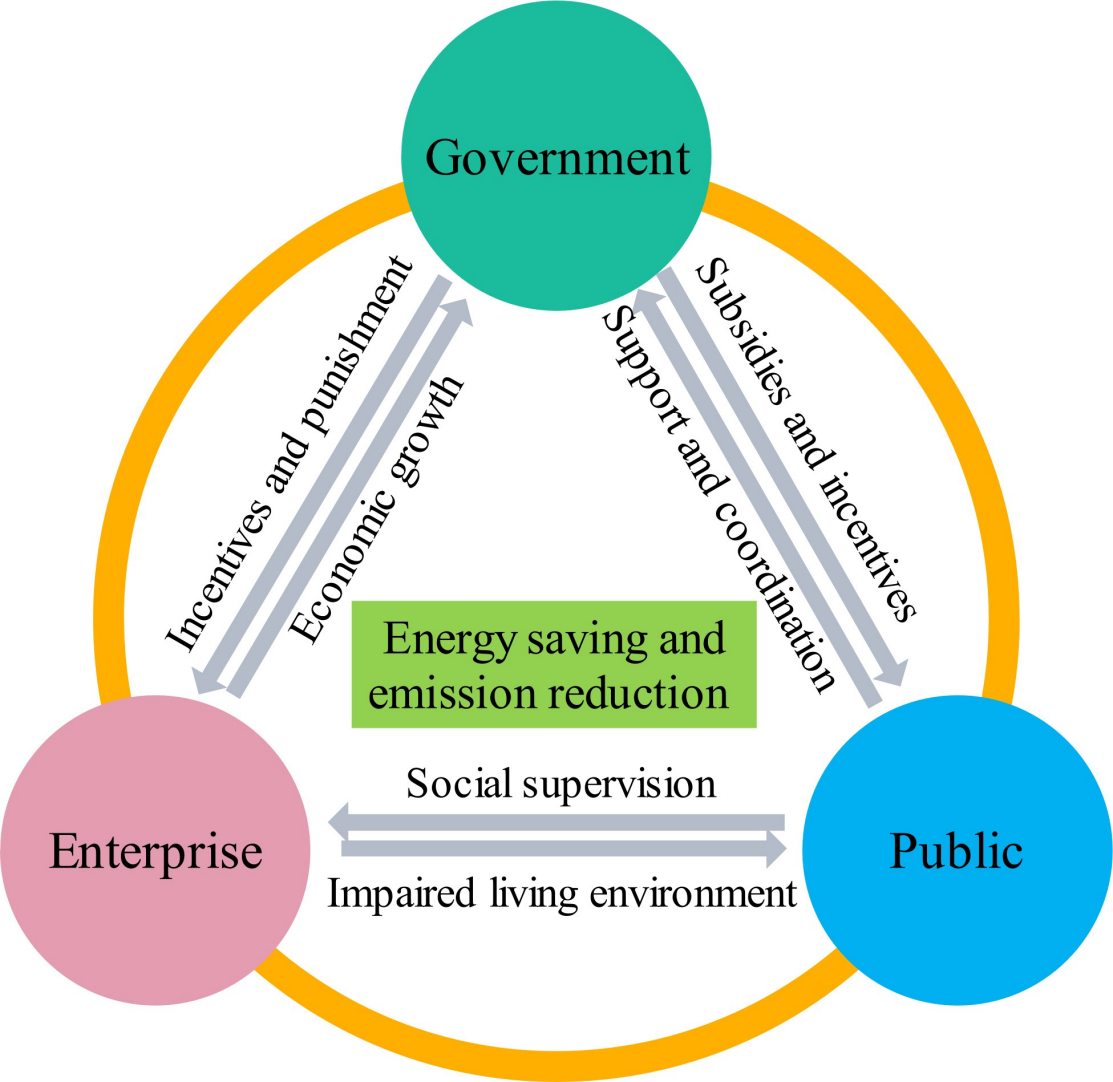
The logical relationships among the three–game players. **Note:** This picture was drawn by the author.

Evolutionary game theory was established by Maynard Smith, and its ideas originated from biological evolution. Unlike traditional game theory, evolutionary game theory only requires the participants of the game to have finite rationality and focuses on the dynamic development process of the game equilibrium [35]. However, evolutionary game theory also faces challenges related to its applicability and inherent drawbacks. It typically relies on the replicator dynamics model, which assumes that the proportion of strategies in a population is constantly changing over time. In situations where the populations are small, discrete, or subject to abrupt changes, replicator dynamics may not accurately capture the dynamics of evolution. Additionally, participants may not fully understand or process all available information when confronted with complex information. They might only process a limited amount of information, ignoring or simplifying other aspects. Since the principles of evolutionary games take into account the benefits of the strategies of the participants and others, the introduction of evolutionary games can better confront the costs and benefits of decision making when challenged by complex and uncertain environments [36]. With the help of evolutionary game theory, this paper links the government, enterprises and the public, and constructs a "government-enterprise-organization" multi-subjects synergistic governance model, which internalizes the externalities between the subjects to improve the governance efficiency. We also analyze the influence of government rewards and punishments on the evolution of energy saving and emission reduction governance through simulation. The logical relationships among the three-game players are shown in Fig 1.

### 3.2. Hypothesis of the model

The game model was constructed to analyze the strategy choices of the game players and examine the stability of the equilibrium point. By setting some parameters to study the equilibrium of interests among different game parties, the specific settings of parameters are shown in Table 1. The assumptions of the model are as follows:

**Hypothesis 1.** The governments are participant 1, the enterprises are participant 2, and the public is participant 3. All participants are limited rationality, and they make corresponding behavioral choices based on their interests and needs. And their strategic choices evolve over time toward optimal decisions.
**Hypothesis 2.** Local governments play a leading role in the process of energy saving and emission reduction, with two behavioral strategy choices: rigorous supervision and loose supervision. When the government chooses to loose supervision, the cost of supervision is *C*_*g*_. Due to the lack of regulation, the local governments may suffer administrative accountability from the central government (-*A*); On the other hand, when the government chooses rigorous supervision, the regulatory cost for the government is *C*_g_*(1+*s*),where *s* represents the proportion of increased costs by local governments under strict regulation. Due to effective regulation, the central government provides financial support (*A*). If the enterprises refuse to adopt energy-efficient production, they will be fined *F*_*e*_ by the local government, in addition to bearing the cost *C* of environmental governance. However, if enterprises actively undertake energy saving and emissions reduction measures, it will bring the government a potential image benefit of *A*_*g*_. Simultaneously, the government will provide tax incentives and reward subsidies, denoted as *M*_*t*_, to manufacturing enterprises. Moreover, local governments will offer corresponding rewards, denoted as *M*_*p*_, for public supervision and reporting of adverse behaviors by enterprises. In this case, the government’s reputation and regulatory image will improve, leading to a reputation benefit (*A*_*s*_). Furthermore, regardless of whether the government chooses to strictly supervision, when the enterprises choose to adopt energy-efficient production, the government will get the synthesized benefits *A*_*g*_, due to the improvement of the environment.
**Hypothesis 3**. For enterprises, the fundamental goal is to maximize their business profits. When the enterprises produce the products for sale, their sales are *R*. During the production process, on the one hand, enterprises may pursue higher profits to reduce the cost of investment in green technology and equipment, which will cause damage to the public (*J*). On the other hand, enterprises may be concerned about government fines and public scrutiny. They may actively cooperate for collaborative governance. This involves introducing green environmental equipment and technologies, transitioning to green production, and striving to minimize energy consumption and emissions. Thus, the production cost will increase from *C*_*p*_ to *C*_*e*_(*C*_*e*_ >*C*_*p*_, and *C*_*e*_-*C*_*p*_>*M*_*t*_).
**Hypothesis 4.** When the public positively participates in supervision, the cost of monitoring is C. If the enterprise does not carry out energy saving and emission reduction causing pollution will need to pay compensation to the public (*F*_*p*_). Similarly, if the public is not engaged in supervision, it will not have access to information on strategic choices from the government. In this case, neither the enterprise nor the government compensates or rewards them.

**Table 1:** Parameters of the tripartite evolutionary game model.

Subject	Parameters	Descriptions
**government**	*C*_*g*_	The cost of loose government supervision
	*A*	The central government’s rewards and penalties for local governments
	*s*	The ratio of increased cost due to strict government regulation
	*F*_*e*_	Fines imposed by the government on enterprises that do not adopt energy-efficient production
	*A*_*s*_	Potential image benefits to the government from the positive monitoring of the public.
	*A*_*g*_	Potential image benefits to the government from the enterprises that adopt energy-efficient production
**enterprises**	*M*_*t*_	Enterprises that adopt energy-efficient production receive tax subsidies from the government. (*F*_*e*_ > *M*_*t*_)
	*G*	Social governance costs paid by enterprises that pollute by not adopting energy-efficient production
	*R*	Enterprise business income
	*C*_*e*_	The costs to be paid by enterprises for adopting energy-efficient production
	*C*_*p*_	The costs to be paid by enterprises for not adopting energy-efficient production (*C*_*e*_ > *C*_*p*_, and *C*_*e*_—*C*_*p*_ > *M*_*t*_)
**public**	*M*_*p*_	Public participation in monitoring receives incentives from the government
	*F*_*p*_	Enterprises do not adopt energy-efficient production, causing pollution and paying compensation to the public.
	*C*	The cost of public monitoring
	*J*	The losses to the public caused by pollution from enterprises that do not adopt energy-efficient production
	*B*	Benefits to the public from energy-efficient production by enterprises

### 3.3. Model analysis

Based on the above assumptions, a tripartite evolutionary game model of "government-enterprise-public" was constructed. Each of the game players has two strategy choices. The set of strategic options for the governments is *M* = {α1,α2} = {rigorous supervision, loose supervision}, and the probability of choosing *α*1 is *x*, while the probability of choosing *α*2 is (1-*x*),*x*∈[0,1]; The set of strategic options for the enterprises is C = {*β*1,*β*2} = {adopt energy-efficient production, not adopt energy-efficient production}, and the probability of choosing *β*1 is *y*, then the probability of choosing *β*2 is (1-*y*),*y*∈[0,1]; The set of strategic options for the public is *K* = {γ1,γ2} = {positive participation, negative participation} and the probability of choosing γ1 is *z*, then the probability of choosing γ1 is (1-*z*),*z*∈[0,1]. In this article, eight possible strategy combinations are shown in Table 2.

**Table 2:** The payoffs matrix among the government, enterprises, and public.

Strategies		Government	Enterprises	Public
(M_1_,C_1_,K_1_)	(*x*,*y*,*z*)	*A*_*g*_ + *A*_*s*_−*M*_*t*_ + *A*−*C*_*g*_(1+*s*)	*R* + *M*_*t*_−*C*_*e*_	*B* + *M*_*p*_—*C*
(M_1_,C_1_,K_2_)	(*x*,*y*,1−*z*)	*A*_*g*_−*M*_*t*_ +*A*–*C*_*g*_ (1+*s*)	*R* + *M*_*t*_−*C*_*e*_	*B*
(M_1_,C_2_,K_1_)	(*x*,1−*y*,*z*)	*F*_*e*_ + *A*_*s*_−*M*_*p*_−*C*_*g*_(1+*s*)+*A*	*R*–*C*_*p*_−*F*_*e*_−*F*_*p*_—*G*	*M*_*p*_ + *F*_*p*_−*C*—*J*
(M_1_,C_2_,K_2_)	(*x*,1−*y*,1−*z*)	*F*_*e*_ + *A*− *C*_*g*_(1+*s*)	*R*–*C*_*p*_−*F*_*e*_—*G*	−*J*
(M_2_,C_1_,K_1_)	(1−*x*,*y*,*z*)	*A*_*g*_−*C*_*g*_*—A*	*R*—*C*_*e*_	*B*–*C*
(M_2_,C_1_,K_2_)	(1−*x*,*y*,1−*z*)	*A*_*g*_−*C*_*g*_*—A*	*R*—*C*_*e*_	*B*
(M_2_,C_2_,K_1_)	(1−*x*,1−*y*,*z*)	−*C*_*g*_—*A*	*R*—*C*_*p*_*—F*_*p*_	*F*_*p*_−*C*—*J*
(M_2_,C_2_,K_2_)	(1−*x*,1−*y*,1−*z*)	−*C*_*g*_—*A*	*R*–*C*_*p*_	−*J*

#### 3.3.1. Evolution stable strategy analysis of government

*E*_11_ represents the expected earning of rigorous supervision by government, while *E*_12_ represents the expected earning of loose supervision by government. $\bar{E_{1}}$ refers to the average expected income.


$$\begin{array}{c} E_{11}=yz\left(A_{g}+A_{s}-M_{t}-C_{g}(1+s)+A\right)+y\left(1-z\right)\left(A_{g}-M_{t}-C_{g}(1+s)+A\right)\\ +\left(1-y\right)z\left(F_{e}+A_{s}-M_{p}-C_{g}(1+s)+A\right)+\left(1-y\right)\left(1-z\right)\left(F_{e}-C_{g}(1+s)+A\right) \end{array}$$ (1)

$$E_{12}=yz(A_{g}-C_{g}-A)+y\left(1-z\right)(A_{g}-C_{g}-A)+(1-y)z(-C_{g}-A)-(1-y)(1-z)(C_{g}+A)$$ (2)

$$\bar{E_{1}}=xE_{11}+\left(1-x\right)E_{12}=2xA-A-C_{\text{g}}+yA_{\text{g}}+xF_{e}-xC_{g}*s+xzA_{s}-xyF_{e}-xyM_{t}-xzM_{p}+xyz*M_{p}$$ (3)

The government’s replicated dynamic equation is as follows:

$$\begin{array}{c} F\left(x\right)=\frac{dx}{dt}=x\left(E_{11}-\bar{E1}\right)=x\left[E_{11}-xE_{11}-\left(1-x\right)E_{12}\right]=x\left(1-x\right)\left(E_{11}-E_{12}\right)\\ =x\left(x-1\right)(s*C_{g}-2A-F_{e}+z(M_{p}-yM_{p}-A_{s})+y(F_{e}+M_{t})) \end{array}$$ (4)

The first-order derivative of *x* and the equation for *G*(*y*) are as follows:

$$\frac{d\left(F\left(x\right)\right)}{dx}=(2x-1)\;(s*C_{g}-2A-F_{e}+z(M_{p}-yM_{p}-A_{s})+y(F_{e}+M_{t}))$$ (5)

$$G\left(y\right)=s*C_{g}-2A-F_{e}+z(M_{p}-yM_{p}-A_{s})+y(F_{e}+M_{t})$$ (6)

**Fig 2:**
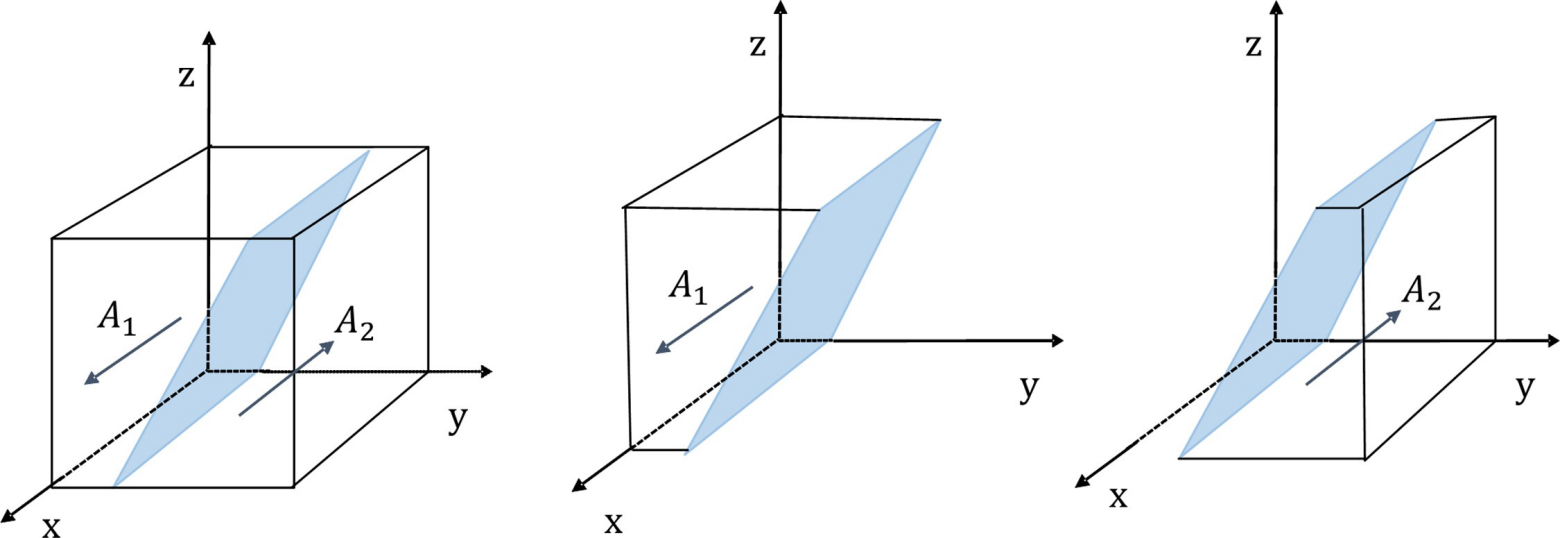
The evolutionary phase diagram of the evolution strategy of the government.

According to the stability theorem for differential equations, in the steady state, the probability that the government chooses to strictly regulate must be satisfied *F*(*x*) = 0 and $\frac{d(F(x))}{dx}<0$.Due to $\frac{\partial G(y)}{\partial y}<0$, *G*(*y*) is an increasing function of *y*. Therefore, when $y=y^{*}=\left(F_{e}+2A-s*C_{g}-z\left(M_{p}-yM_{p}-A_{s}\right)\right)/\left(F_{e}+M_{t}\right)$,which make *G*(*y*) = 0, then $\frac{d(F(x))}{dx}\equiv 0$, and the government can’t identify the stabilization strategy; When *y* < y* its with *G*(*y*) < 0, which implies that $\frac{d\left(F(x)\right)}{dy}{|}_{x=1}<0,\frac{d\left(F(x)\right)}{dx}{|}_{x=0}>0$, so *x* = 1 is the evolutionary stabilization strategy (ESS). Conversely, when *y* > y*, *x* = 0 is the ESS point. In this case, the government chooses the loose regulatory strategy. The phase diagram of the evolution strategy of the government is shown in Fig 2.

In Fig 2, the probability that the governments adopt rigorous supervision is the volume of *A*_1_, and the probability that the government loose supervision is the volume of *A*_2_. The size of its volumes is closely related to y^*^. With the other parameters kept constant, if the value of *C*_*g*_,*F*_*e*_, *s*,*M*_*p*_ and *M*_*t*_ respectively increased, it will result in a decrease in y^*^. In this case, the volume of *A*_1_ will decrease, while the volume of *A*_2_ will increase, indicating that the government will be more likely to adopt a strategy of strict supervision; When the value of *A* and *A*_*s*_ increase, it leads to an increase in y^*^. In this case, the volume of *A*_1_ will increase, while the volume of *A*_2_ will decrease, which shows that the governments will adopt a strategy of strict supervision. From this, we can draw two conclusions:

**Conclusion 1.** Local governments’ strategic choices are influenced by the behavioral strategies of the enterprises and the public. The government’s intensity of strict supervision is positively correlated with the central government’s rewards and penalties(*A*), the local governments fines to enterprises (*F*_*e*_), and the incentives that the public participation in monitoring receives from the government (*A*_*s*_); It is negatively correlated with the following variables: local governments reward for the public’s supervisory behavior (*M*_*p*_), the tax benefits that the governments reward for enterprises (*M*_*t*_), and the ratio of increased cost due to strict government regulation (*s*). This shows that when the local government increases the number of rewards and punishments, it can enhance the willingness of the enterprises and the public to participate in energy saving and emission reduction. At this time, the local governments are more inclined to choose a loose supervision strategy. This shows that the government can not only enhance the level of participation by improving rewards and penalties for enterprises and the public, but also allocate administrative resources based on societal needs and issue priorities.
**Conclusion 2.** In the evolutionary process, the central government’s incentives and administrative penalties can effectively eliminate the phenomenon of local governments’ loose supervision. In addition, the potential image benefits for local governments brought by the public’s cooperation will also incentivize local governments to adopt strict supervision. However, an increase in local government regulatory costs can further burden local finances, which is not conducive to the fulfillment of local government responsibilities.

#### 3.3.2. Evolution stable strategy analysis of enterprise

*E*_21_ represents the expected earning of not adopting energy-efficient production by enterprises, while *E*_22_ represents the expected earning of adopting energy-efficient production by enterprises. $\bar{E_{2}}$refers to the average expected income of enterprises.


$$\begin{array}{c} E_{21}=xz\left(R+M_{t}-C_{e}\right)+x\left(1-z\right)\left(R+M_{t}-C_{e}\right)\\ +\left(1-x\right)z\left(R-C_{e}\right)+\left(1-x\right)\left(1-z\right)\left(R-C_{e}\right) \end{array}$$ (7)

$$\begin{array}{c} E_{22}=xz\left(R-C_{p}-F_{e}-F_{p}-G\right)+x\left(1-z\right)\left(R-C_{p}-F_{e}-G\right)\\ +\left(1-x\right)z\left(R-C_{p}-F_{p}\right)+\left(1-x\right)\left(1-z\right)\left(R-C_{p}\right) \end{array}$$ (8)

$$\bar{E2}=yE_{21}+\left(1-y\right)E_{22}=R-C_{p}-yC_{e}+yC_{p}-xF_{e}-xG-zF_{p}+xyF_{e}+xyG+yzF_{p}+xyM_{t}$$ (9)

The enterprise’s replicated dynamic equation is as follows:

$$\begin{array}{c} F\left(y\right)=\frac{dy}{dt}=y\left(E_{21}-\bar{E2}\right)=y\left[E_{21}-y*E_{21}-\left(1-y\right)*E_{22}\right]\\ =y\left(1-y\right)\left(E_{21}-E_{22}\right)=y\left(y-1\right)\left(C_{e}-C_{p}-x(F_{e}+M_{t}+G)-zF_{p}\right) \end{array}$$ (10)

The first-order derivative of *y* and the equation for *H*(*x*) are as follows:

$$\frac{d\left(F\left(y\right)\right)}{dy}=\left(2y-1\right)\left(C_{e}-C_{p}-x(F_{e}+M_{t}+G)-zF_{p}\right)$$ (11)

$$H\left(x\right)=C_{e}-C_{p}-x(F_{e}+M_{t}+G)-zF_{p}$$ (12)

**Fig 3:**
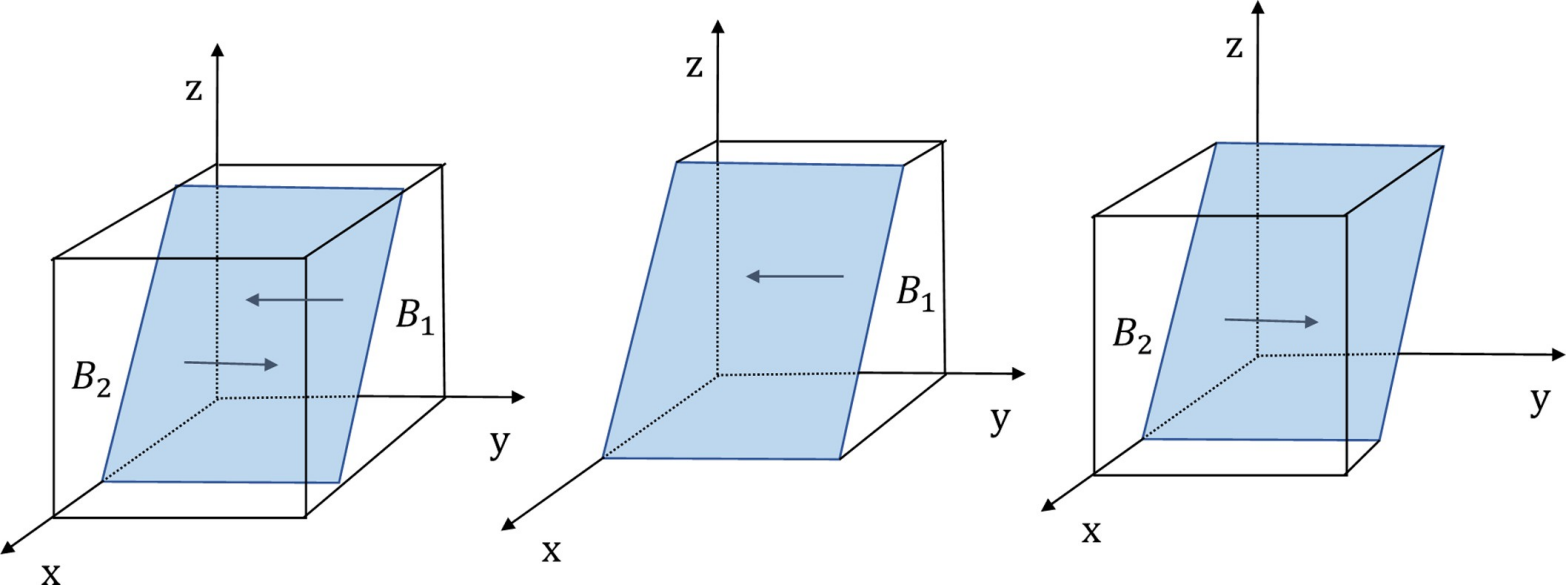
The evolutionary phase diagram of the evolution strategy of the enterprises.

According to the stability theorem for differential equations, in the steady state, the probability that the enterprises choose to adopt energy efficient must be satisfied *F*(*y*) = 0 and $\frac{d(F\left(y\right)}{dy}<0$. Due to $\frac{\partial H\left(x\right)\;}{\partial x}<0$, *H*(*x*) is an increasing function of *x*. So, when $x=x^{*}=\left(Ce-Cp-zFp\right)/\left(Fe+Mt+G\right),$ which makes *H*(*x*) = 0, then $\frac{d\left(F\left(y\right)\right)}{dy}\equiv 0$, the enterprise can’t identify the stabilization strategy; when *x* < x*, *H*(*x*) > 0 then $\frac{d(F\left(y\right)}{dy}{|}_{y=0}<0$, so *y* = 0 is the evolutionary stabilization strategy (ESS). Conversely, when *x* < x*, *y* = 1 is the ESS point. The phase diagram of the evolution strategy of the government is shown in Fig 3.

In Fig 3, the probability that enterprises don’t adopt energy-efficient production is the volume of *B*_1_, and the probability that enterprises adopt energy-efficient production is the volume of *B*_2_. The size of its volume is closely related to x*. According to the equilibrium stability point condition $x=x^{*}=\left(C_{e}-C_{p}-zF_{p}\right)/\left(F_{e}+M_{t}+G\right)$, it can be observed that when *C*_*e*_ is increased, it will result an increase in x*. At this point, the volume of *B*_1_ will increase, while the volume of *B*_2_ will decrease, which indicates that the probability of the enterprises don’t adopt energy-efficient production will increase; When the value of *C*_*p*_,*F*_*p*_,*F*_*e*_,*M*_*t*_ and *G* increase, it leads to an decrease in $x=x^{*}=\left(C_{e}-C_{p}-zF_{p}\right)/\left(F_{e}+M_{t}+G\right)$. In this case, the volume of *B*_1_ will decrease and the volume of *B*_2_ will increase, which shows that the probability that the enterprises adopt energy-efficient production will increase. There are two conclusions as follows:

**Conclusion 3.** Enterprise’s strategic choices are positively correlated with the following factors: the production cost paid by enterprises for not adopting energy-efficient production (*C*_*p*_), government fines to enterprises (*F*_*e*_), enterprises that adopt energy-efficient production receive tax subsidies from the government (*M*_*t*_) as well as the social governance costs paid by enterprises that pollute by not adopting energy-efficient production (*G*). It’s negatively correlated with the production costs to be paid by enterprises for adopting energy-efficient production (*C*_*e*_).
**Conclusion 4**. Firstly, in the evolutionary process, the heavier the government’s tax incentives and penalties for enterprises, the higher the probability that the enterprises will adopt energy saving and emission reduction strategies. Secondly, when the cost of not adopting energy-efficient production increases, enterprises will be more inclined to choose energy-efficient production strategies. This illustrates that potential image benefits resulting from energy-efficient production and the implementation of local government’s reward and penalty policies will influence enterprises’ strategic choices. Finally, when the income from not adopting energy-efficient production is less than the benefit brought by energy-efficient production, the enterprises will increase their willingness to participate in governance cooperation. Therefore, local governments can increase incentives and penalties to enhance the willingness of enterprises to collaborate in governance.

#### 3.3.3. Evolution stable strategy analysis of public

*E*_31_ represents the expected earning of positive participation in supervision by the public, while *E*_32_ represents the expected earning of negative participation in supervision by the public. $\bar{E_{3}}$refers to the average expected income.


$$\begin{array}{c} E_{31}=xy\left(B+M_{p}-C\right)+x\left(1-y\right)\left(M_{p}+F_{p}-C-J\right)\\ +\left(1-x\right)y\left(B-C\right)+\left(1-x\right)\left(1-y\right)\left(F_{p}-C-J\right) \end{array}$$ (13)

$$E_{32}=xyB-x\left(1-y\right)J+\left(1-x\right)yB-\left(1-x\right)\left(1-y\right)J$$ (14)

$$\bar{E3}=zE_{31}+\left(1-z\right)E_{32}=yB-J-zC+zF_{p}+yJ-yzF_{p}+xzM_{p}$$ (15)

The public’s replicated dynamic equation is as follows

$$\begin{array}{c} F\left(z\right)=\frac{dz}{dt}=z\left(E_{31}-\bar{E3}\right)=z\left[E_{31}-zE_{31}-\left(1-z\right)E_{32}\right]\\ =z\left(1-z\right)\left(E_{31}-E_{32}\right)=z\left(z-1\right)\left(C-F_{p}+yF_{p}-xM_{p}\right)\; \end{array}$$ (16)

The first-order derivative of *z* and the equation for *L*(*x*) are as follows:

$$\frac{d\left(F\left(z\right)\right)}{dz}=\left(2z-1\right)\left(C-Fp+yFp-xMp\right)\;$$ (17)

$$L\left(x\right)=C-F_{p}+yF_{p}-xM_{p}$$ (18)

**Fig 4:**
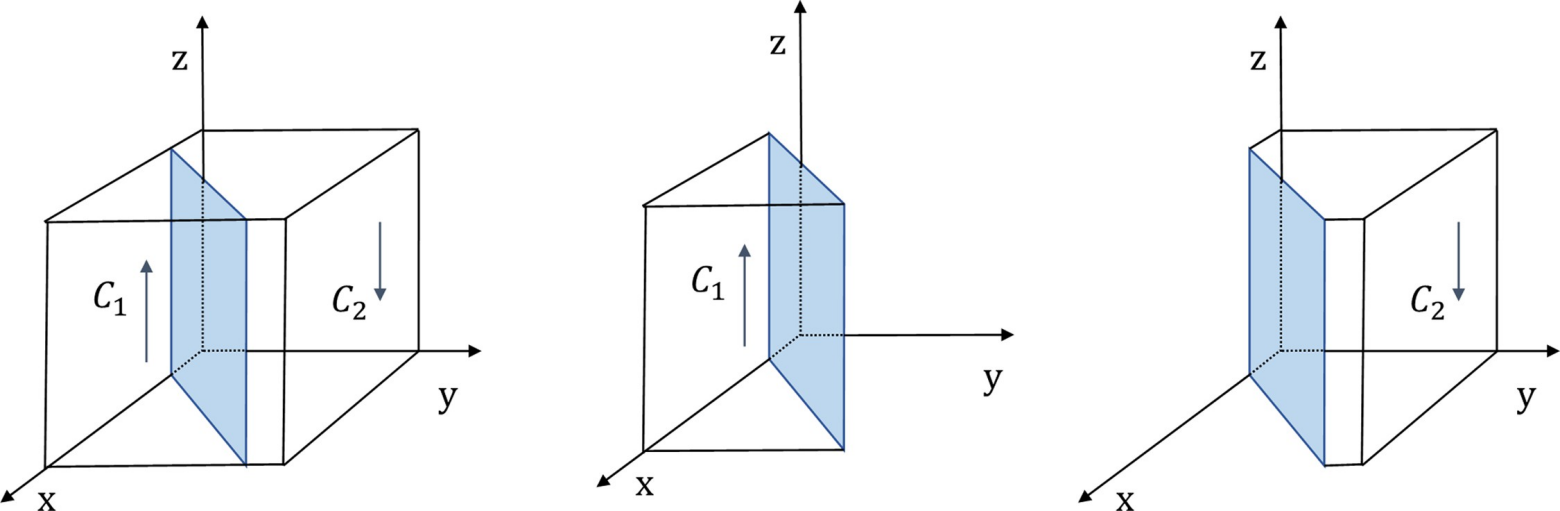
The evolutionary phase diagram of the evolution strategy of the public.

According to the stability theorem for differential equations, in the steady state, the probability that the public chooses to positive participation must be satisfied *F*(*z*) = 0 and $\frac{d(F(z))}{dz}<0$. Due to $\frac{\partial L(x)}{\partial x}<0$, *L*(*x*) is a decreasing function of *x*. Therefore, when $x=x^{*}=\left(Ce-Cp-zFp\right)/\left(Fe+Mt+G\right),$ which makes *L*(*x*) = 0, then $\frac{d\left(F(z)\right)}{dz}\equiv 0$, the public can’t identify the stabilization strategy; When *x* < x**, *L*(*x*) > 0., which implies that $\frac{d\left(F(z)\right)}{dz}{|}_{z=0}<0$, so *z* = 0 is the evolutionary stabilization strategy (ESS). Conversely, when *x* < x**, *z* = 1is the ESS point. The phase diagram of the evolution strategy of the government is shown in Fig 4.

In Fig 4, the probability that the public chooses the positive participation strategy is the volume of *C*_1_, and the probability that the public chooses the negative participation strategy is the volume of *C*_2_. The size of its volume is closely related to the equilibrium point’s condition x**. With other parameters kept constant, according to the condition $x=x^{**}=\frac{C-F_{p}+yF_{p}}{M_{p}}$, if the value of *C* increase, it results in an increase in x**. In this case, the volume of *C*_1_ will decrease and the volume of *C*_2_ will increase, which indicates that the probability that the public adopts the monitoring strategy will decrease; When the value of *M*_*p*_ and *F*_*p*_ increase, such that $x=x^{**}=\frac{C-F_{p}+yF_{p}}{M_{p}}$ decrease, and then the volume of *C*_1_ will increase and the volume of *C*_2_ will decrease, which shows that the probability of the public adopts negative participation strategy will increase. There are two conclusions as follows:

**Conclusion 5.** The public’s strategic choices are positively correlated with the cost that the public monitoring the enterprise (*C*). It is negatively correlated with the compensation to the public for pollution caused by enterprises that choose not to adopt energy-efficient production (*F*_*p*_) and the incentives that the public received from the government by participating in monitoring (*M*_*p*_).

**Conclusion 6.** In the evolutionary process, the heavier the cost of governance paid by the public, the lower the probability that they will participate in collaborative governance. Therefore, the governments can incentivize the public to actively participate in energy-saving and emission reduction governance by appropriately increasing the amount of rewards for the public

### 3.4. Equilibrium point property analysis

By solving the replicated dynamic equations of the government, enterprises and the public, the 16 equilibrium points were obtained as follows: *E*_1_(*x*_1_,0,*z*_1_), *E*_2_(*x*_2_,*y*_1_,1), *E*_3_(0,0,0), *E*_4_(1,0,0), *E*_5_(0,1,0), *E*_6_(0,0,1), *E*_7_(1,1,0), *E*_8_(1,0,1), *E*_9_(0,1,1), *E*_10_(1,1,1), *E*_11_(1,*y*_2_,*z*_2_), *E*_12_(*x*_3_,*y*_3_,*z*_3_), *E*_13_(0,*y*_4_,*z*_4_), *E*_14_(*x*_4_,*y*_5_,*z*_5_), *E*_15_(*x*_5_,1,*z*_6_), *E*_16_(*x*_6_,*y*_6_,0). Then, the Jacobian matrix would be constructed and analyzed for stability and strategy combinations.


$$J=\left[\begin{array}{ccc} J_{1} & J_{2} & J_{3}\\ J_{4} & J_{5} & J_{6}\\ J_{7} & J_{8} & J_{9} \end{array}\right]=\left[\begin{array}{ccc} \frac{\partial F\left(x\right)}{\partial x} & \frac{\partial F\left(x\right)}{\partial y} & \frac{\partial F\left(x\right)}{\partial z}\\ \frac{\partial F\left(y\right)}{\partial x} & \frac{\partial F\left(y\right)}{\partial y} & \frac{\partial F\left(y\right)}{\partial z}\\ \frac{\partial F\left(z\right)}{\partial x} & \frac{\partial F\left(z\right)}{\partial y} & \frac{\partial F\left(z\right)}{\partial z} \end{array}\right]$$

$$=\left[\begin{array}{ccc} \begin{array}{c} (2x-1)(s*C_{g}-F_{e}-2A-zA_{s}\\ +yF_{e}+yM_{t}+zM_{p}+yz*M_{p}) \end{array} & x(x-1)(F_{e}+M_{t}-zM_{p}) & x(x-1)(M_{p}-A_{s}-yM_{p})\\ -y(y-1)(F_{e}+M_{t}+G) & \begin{array}{c} -(2y-1)(C_{p}-C_{e}+xF_{e}\\ +xG+zF_{p}+xM_{t}) \end{array} & -y(y-1)F_{p}\\ z(1-z)M_{p} & z(z-1)F_{p} & \left(2z-1)(C-F_{p}+yF_{p}-xM_{p}\right) \end{array}\right]$$

According to Lyapunov’s first method: when every eigenvalue in the matrix is negative, then the equilibrium point is asymptotically stable; when there is a positive value for at least one of the eigenvalues, then the equilibrium point is unstable; If eigenvalues, other than zero, are all negative, the stability of the equilibrium point is uncertain [37, 38]. The 16 equilibrium points were brought into the Jacobian matrix, and then obtained the eigenvalues and stability of the equilibrium points, the details were shown in Table 3.

**Table 3:** Equilibrium point analysis.

Equilibrium point	Eigenvalue	Symbol	ESS
*E*_1_(*x*_1_,0,*z*_1_)	α_1_,α_2_,-α_1_	(+,x,-)	Uncertain
*E*_2_(*x*_2_,*y*_1_,1)	α_3_,α_4_,-α_4_	(x,+,-)	Uncertain
*E*_3_(0,0,0)	*C*_*p*_-*C*_*e*_,*F*_*p*_-*C*,2*A* + *F*_*e*_-s*C_*g*_	(-,+,+)	Unstable
*E*_4_(1,0,0)	s*C_*g*_—*F*_*e*_-2*A*,*F*_*p*_-*C*+*M*_*p*_,*C*_*p*_-*C*_*e*_+*F*_*e*_+G+*M*_*t*_	(-,+,+)	Unstable
*E*_5_(0,1,0)	*C*_*e*_-*C*_*p*_,-*C*,2*A* − *M*_*t*_-s*C_*g*_	,-,+)	Unstable
*E*_6_(0,0,1)	*C*—*F*_*e*,_*C*_*p*_*−C*_*e*_ + *F*_*p*_, 2*A*+*A*_*s*_+*F*_*e*_−*M*_*p*_−s*C_*g*_	(-,-,+)	Unstable
*E*_7_(1,1,0)	*M*_*p*_−*C*,*M*_*t*_− 2*A* + s*C_*g*_,*C*_*e*_−*C*_*p*_−*F*_*e*_−*G*—*M*_*t*_	(+,-,-)	Unstable
*E*_8_(1,0,1)	*C*–*F*_*b*_−*M*_*p*_,*M*_*p*_−*A*_*s*_−*F*_*e*_− 2*A* + s* *C*_*g*_,*C*_*p*_−*C*_*e*_ + *F*_*e*_ + *F*_*p*_ + *G* +*M*_*t*_	(-,-,+)	Unstable
*E*_9_(0,1,1)	*C*,*C*_*e*_−*C*_*p*_−*F*_*p*_,2*A* + *A*_*s*_−*M*_*t*_−s* *C*_*g*_	(+,x,+)	Uncertain
*E*_10_(1,1,1)	*C*–*M*_*p*_,*M*_*t*_—*A*_*s*_ 2*A* + s*C_*g*_,*C*_*e*_—*C*_*p*_−*F*_*e*_−*F*_*p*_−*G*—*M*_*t*_	(-,-,-)	ESS
*E*_11_(1,*y*_2_,*z*_2_)	α_5_,-α_5_,α_6_	(+,-,x)	Uncertain
*E*_12_(*x*_3_,*y*_3_,*z*_3_)	α_7_,α_8_,α_9_	(x,+,-)	Uncertain
*E*_13_(0,*y*_4_,*z*_4_)	α_10_,α_11_,-α_11_	(x,+,-)	Uncertain
*E*_14_(*x*_4_,*y*_5_,*z*_5_)	α_12_,α_13_,α_14_	(+,-,x)	Uncertain
*E*_15_(*x*_5_,1,*z*_6_)	α_15_,-α_15_,α_16_	(+,-,x)	Uncertain
*E*_16_(*x*_6_,*y*_6_,0)	α_17_,α_18_,-α_17_	(+,x,-)	Uncertain

* In the above analyses, a number of assumptions should be made: (1) *A* + *F*_*e*_−s*C_*g*_ > 0, (2) *C*–*M*_*p*_ < 0, (3) *C*_*e*_−*C*_*p*_−*F*_*e*_−*F*_*p*_—*G*–*M*_*t*_ < 0, (4) *F*_*p*_−*C* > 0, (5) *M*_*t*_−*As*– 2*A* + s* *C*_*g*_ < 0 x_*,_y_*,_z_*_are the axes of the equilibrium points, and the *x* symbol expresses uncertainty.

**Conclusion 7.** When conditions (2), (3) and (5) are satisfied, there is one stable point *E*_10_(1,1,1), which indicated that the strategic choices of enterprises and the public are related to the reward and punishment strategies of local governments. In addition, the strategic choices of local governments are highly correlated with the policies support and administrative accountability of the central government. According to the above constraints and combined with the previous assumptions, it can be inferred that when the benefits obtained by the local governments exceed the regulatory costs, the strategy choice of the local governments will eventually evolve to strict supervision. Thus, it can be seen that the incentive and punishment mechanisms can influence ethical risk behavior. And the decisions of the game players are strongly related to their respective relative returns. Therefore, in a market with information asymmetry, the government, by establishing penalty mechanisms, ensures that the cost savings of production enterprises for not implementing energy saving production are significantly lower than the rewards and penalties they receive. In such a scenario, enterprises will gradually evolve towards energy-efficient production strategies. Similarly, when the rewards for public participation in supervision outweigh the costs involved, their strategy choices will also evolve toward participating in supervision.

### 3.5. Robustness tests

To provide a more intuitive representation of the dynamic evolution of strategies in the "Government-Enterprise-Social Public" model, numerical simulation and modeling were conducted using Matlab R2016b software. The relevant parameters were assigned values by drawing on the practice of other scholars [34, 39, 40]. The initial values of each parameter in the model are set as shown in Table 4. These initial values provide a foundation for the dynamic simulation of the model. Further adjustments or sensitivity analyses can be conducted to explore the model’s behavior under different scenarios.

**Table 4:** The initial set of values of parameters in the numerical simulation.

Parameters	*C*_*g*_	*M*_*t*_	*M*_*p*_	*A*	*F*_*e*_	*G*	*s*	*A*_*s*_	*C*_*e*_	*C*_*p*_	*C*	*F*_*p*_
**Values**	100	30	30	50	30	40	0.2	20	200	150	10	20

**Fig 5:**
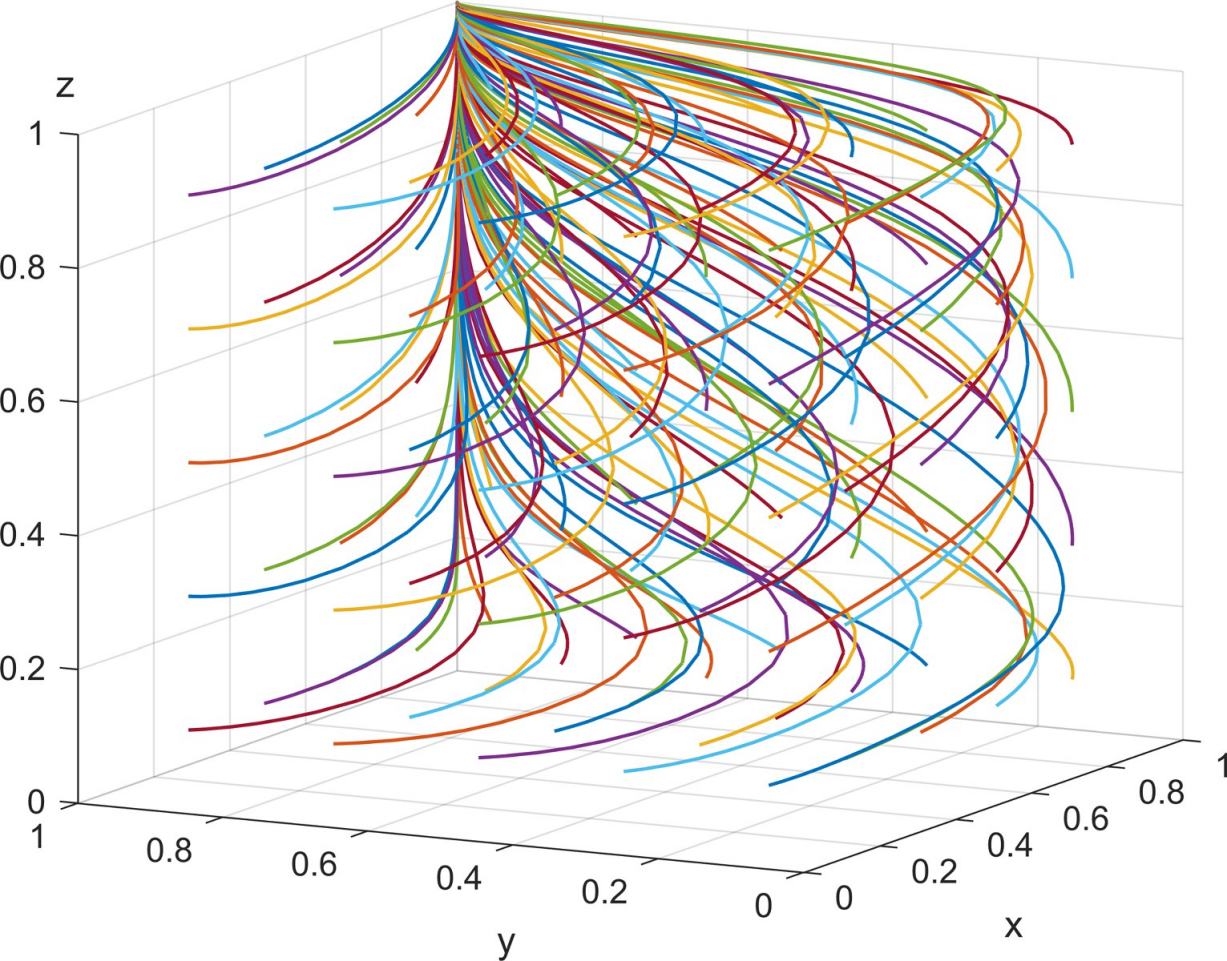
Robustness tests for equilibrium points.

When the parameter values are set to the initial values, the simulation results after replicating the dynamic equations 50 times. There are 50 solid lines with evolution directions in Fig 5, representing the evolution of 50 strategy combinations over time. We can see that the initial strategy combinations are evenly distributed in the cube, and the result is that they all evolve to the equilibrium *E*10(1,1,1). This shows that the government adopting rigorous supervision, enterprises adopting energy-efficient production, and the public positive participation in supervision can achieve the optimal state. They are adopted to demonstrate the feasibility of *E*10(1,1,1) as the unique ESS and the validity of the pattern of coordinated governance. The simulation results are consistent with the findings of the evolutionary stability analysis of the system, which indicates that the model setup is reasonable.

## 4. Results and discussion

### 4.1. The analysis of regulatory costs

**Fig 6:**
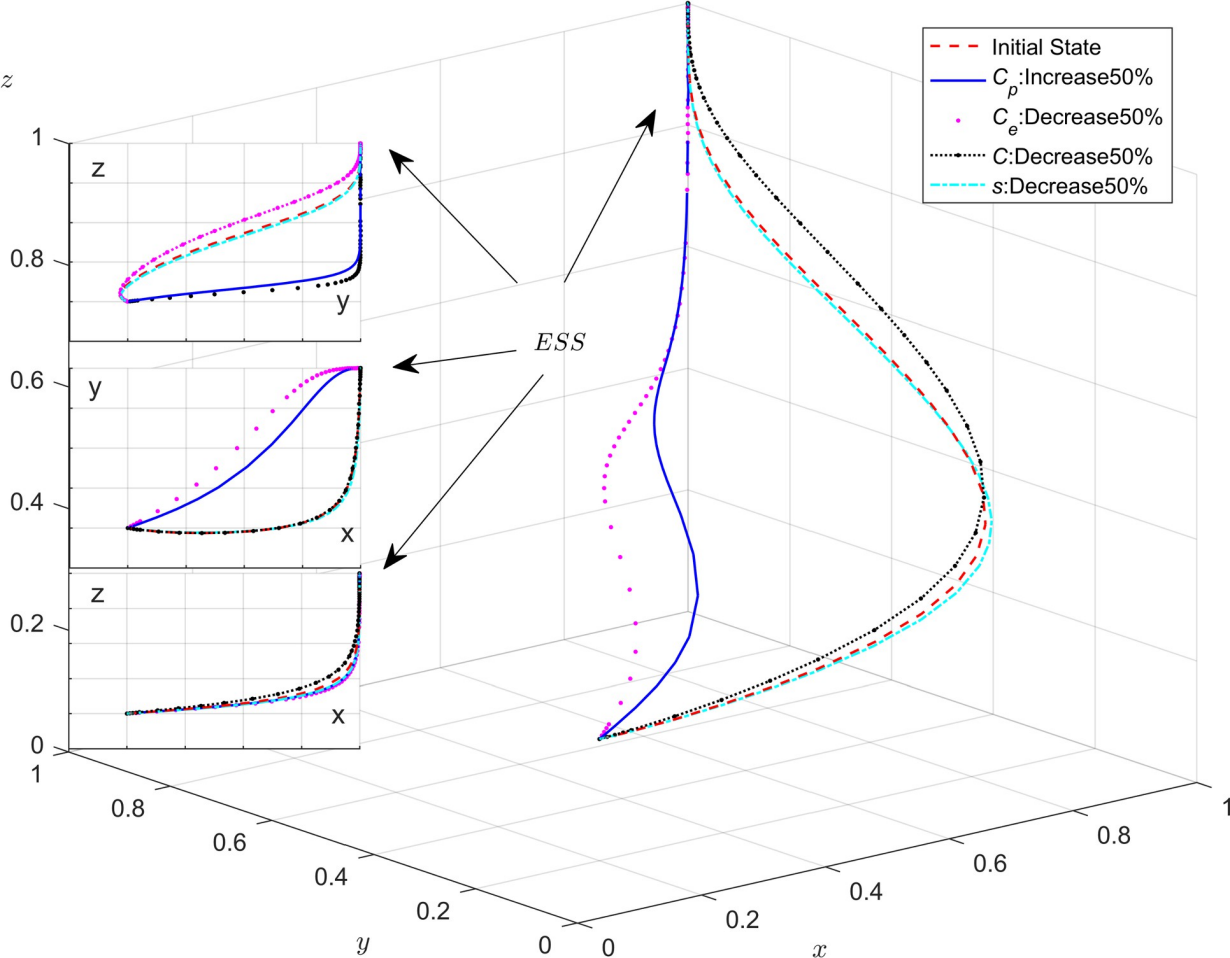
Impact of regulatory costs on tripartite evolutionary trends.

Combining with Table 3 and conclude that the optimal ESS is a stable equilibrium with strict government regulation and active public participation in monitoring, which is conducive to the green transformation of enterprises. Therefore, we aim to make E_10_(1,1,1) an ESS. We noted that the system has a stabilization point E_10_(1,1,1) when conditions $C-M_{p}<0,C_{e}-C_{p}-F_{e}-F_{p}-G-M_{t}<0,\;\text{and}\;M_{t}-A_{s}-2A+s*C_{g}<0$are satisfied. Thus, under these conditions, adjusting the parameter values of regulatory costs makes it possible to observe how changes in regulatory costs affect the behavior of the players during the evolutionary game. To ensure $C-M_{p}<0,C_{e}-C_{p}-F_{e}-F_{p}-G-M_{t}<0,\;\text{and}\;M_{t}-A_{s}-2A+s*C_{g}<0,$ the values of parameters ***s***, ***C***_***e***_, ***C***_***p***_, and *C* need to be reasonable, which are shown in Table 5. In order to better present the simulation results in this paper, we used Matlab software for simulation. We bring the values from Table 5 into the model for simulation, and the specific results are shown in Fig 6.

**Table 5:** The set of values of parameters in the numerical simulation of regulatory costs.

Parameters	*s*	*C*_*e*_	*C*_*p*_	*C*
**Initial Values**	0.2	200	150	10
**Changed Values**	0.1	100	225	5

The positive variable *C*_*p*_ was increased by 50% and the values of the negative variables *C*_*e*_, *C* and *s* were individually decreased by 50%. For enterprises, the evolutionary rate is strongly affected by the cost, especially the cost of production. Under strict government regulation, as the system evolves towards stability, reducing the cost of introducing advanced technologies and equipment can accelerate the rate at which production enterprises adopt energy saving and emission reduction strategies. In this scenario, the probability of production enterprises adopting energy saving and emission reduction strategies will rise; For governments, we can conclude that the variable of *C*_*p*_ has a negligible effect on the choice of government strategy. Conversely, the variable *C* has a strong effect on the public’s choice of strategy, which means that the cost of regulation is reduced, and the probability of public participation in monitoring will be higher.

### 4.2. The analysis of penalties

To analyze the sensitivity of the penalty, the parameter values in Table 6 were substituted into this model to obtain the evolutionary simulation results. And the method is similar to 4.1.

**Table 6:** The set of values of parameters in the numerical simulation of penalties.

Parameters	*A*	*F*_*e*_	*G*	*F*_*p*_
**Initial Values**	50	30	40	20
**Changed Values**	30	10	10	10
	70	50	70	30

**Fig 7:**
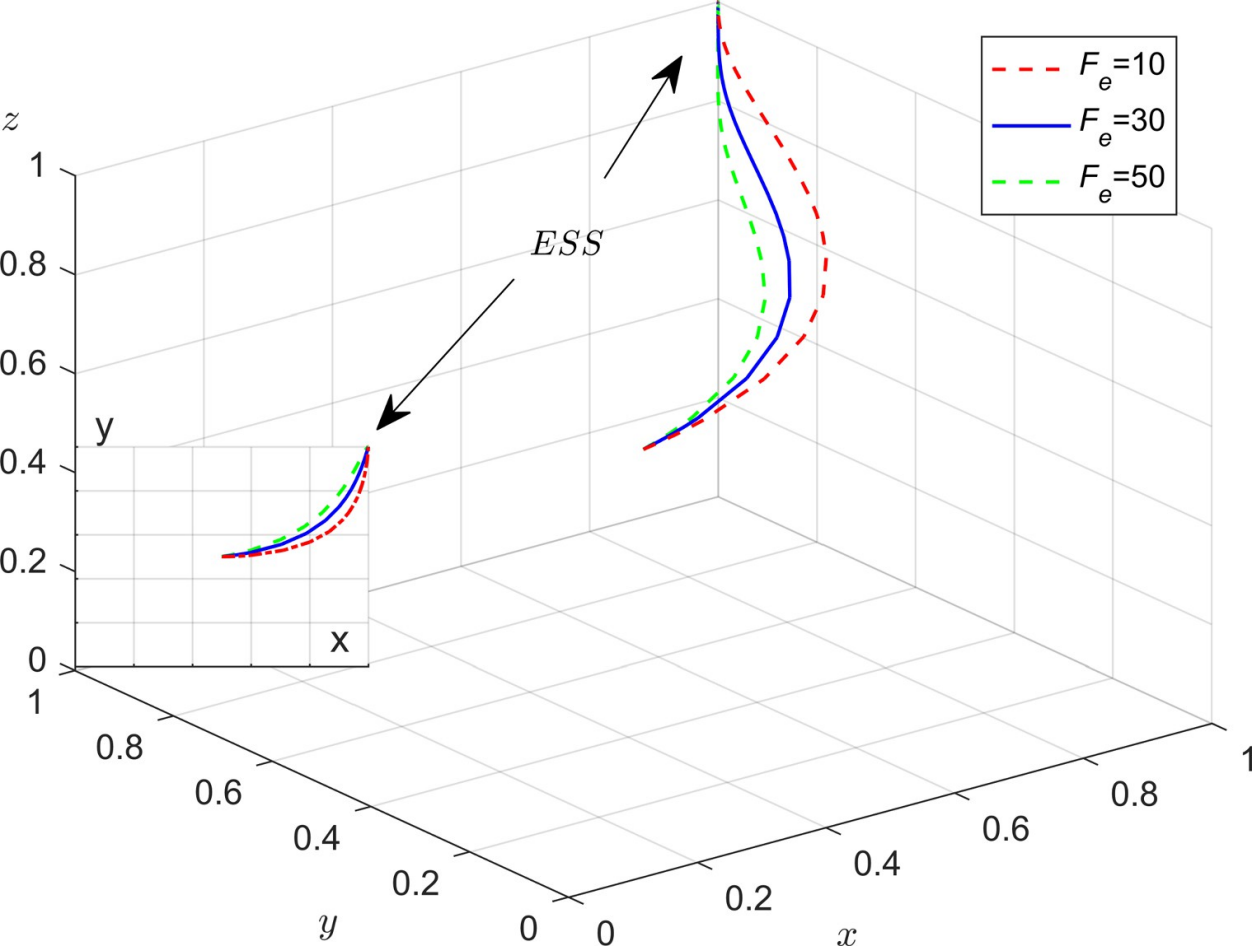
Impact of government fines on enterprises.

**Fig 8:**
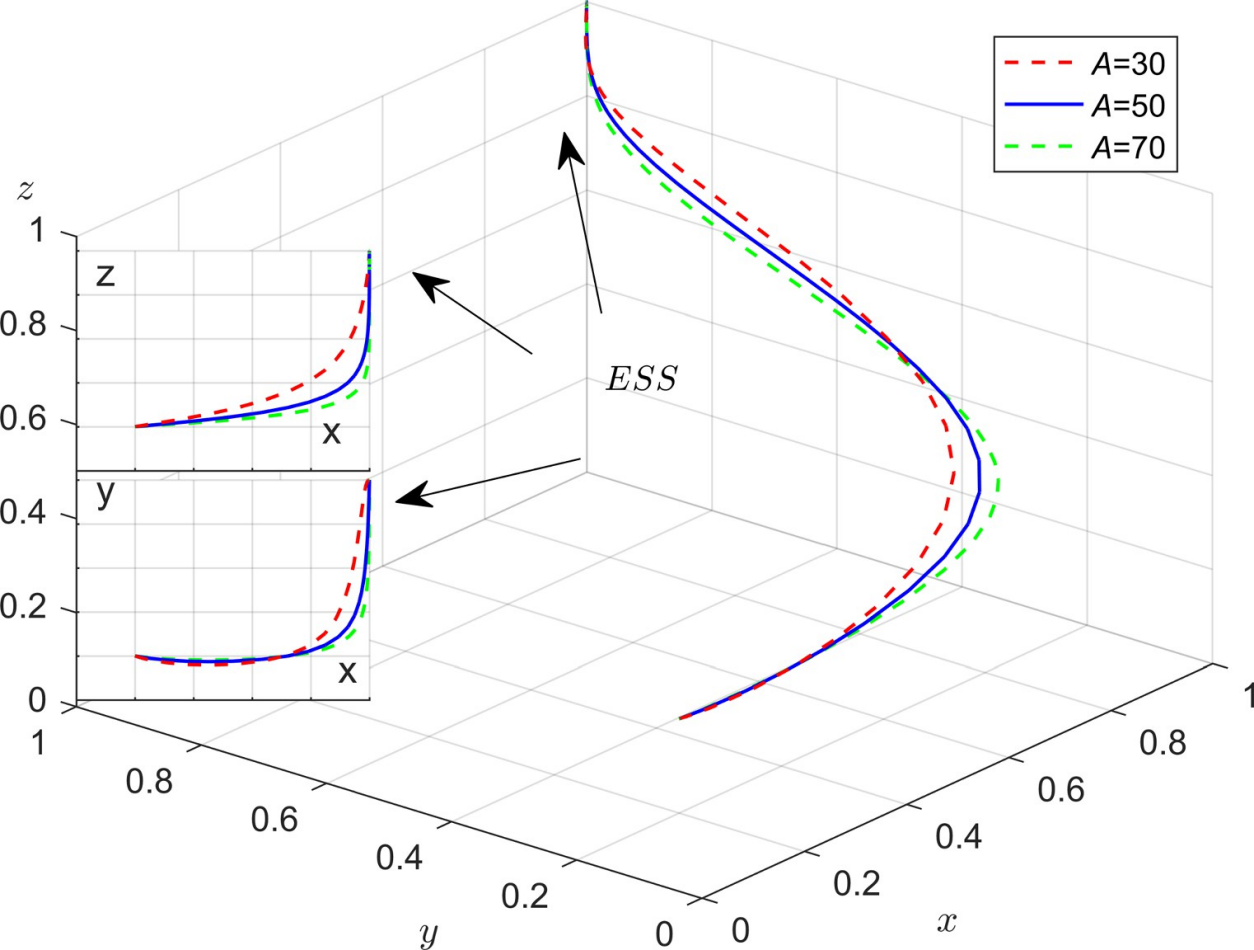
The impact of the central government’s rewards and penalties on the local government.

**Fig 9:**
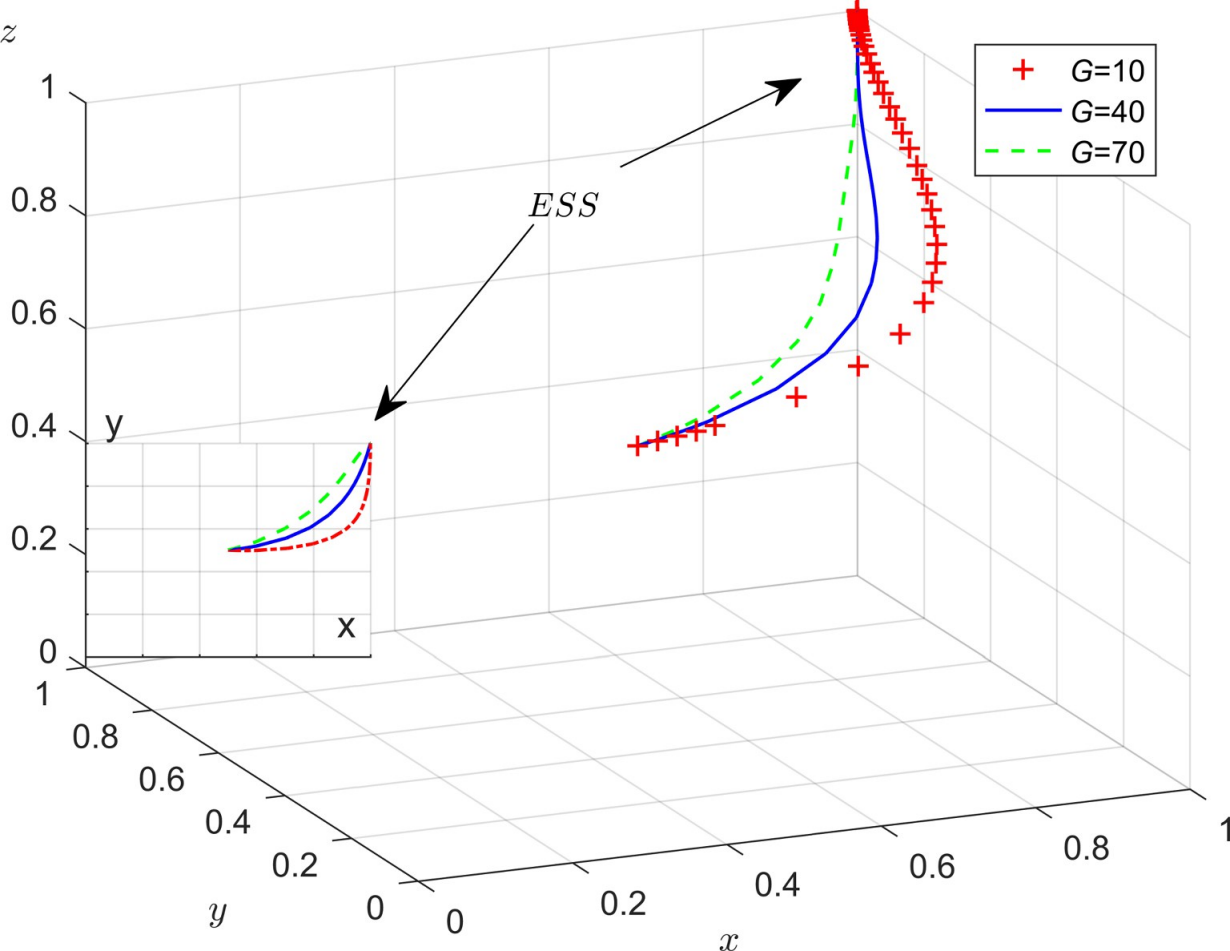
The cost of social governance to enterprises.

**Fig 10:**
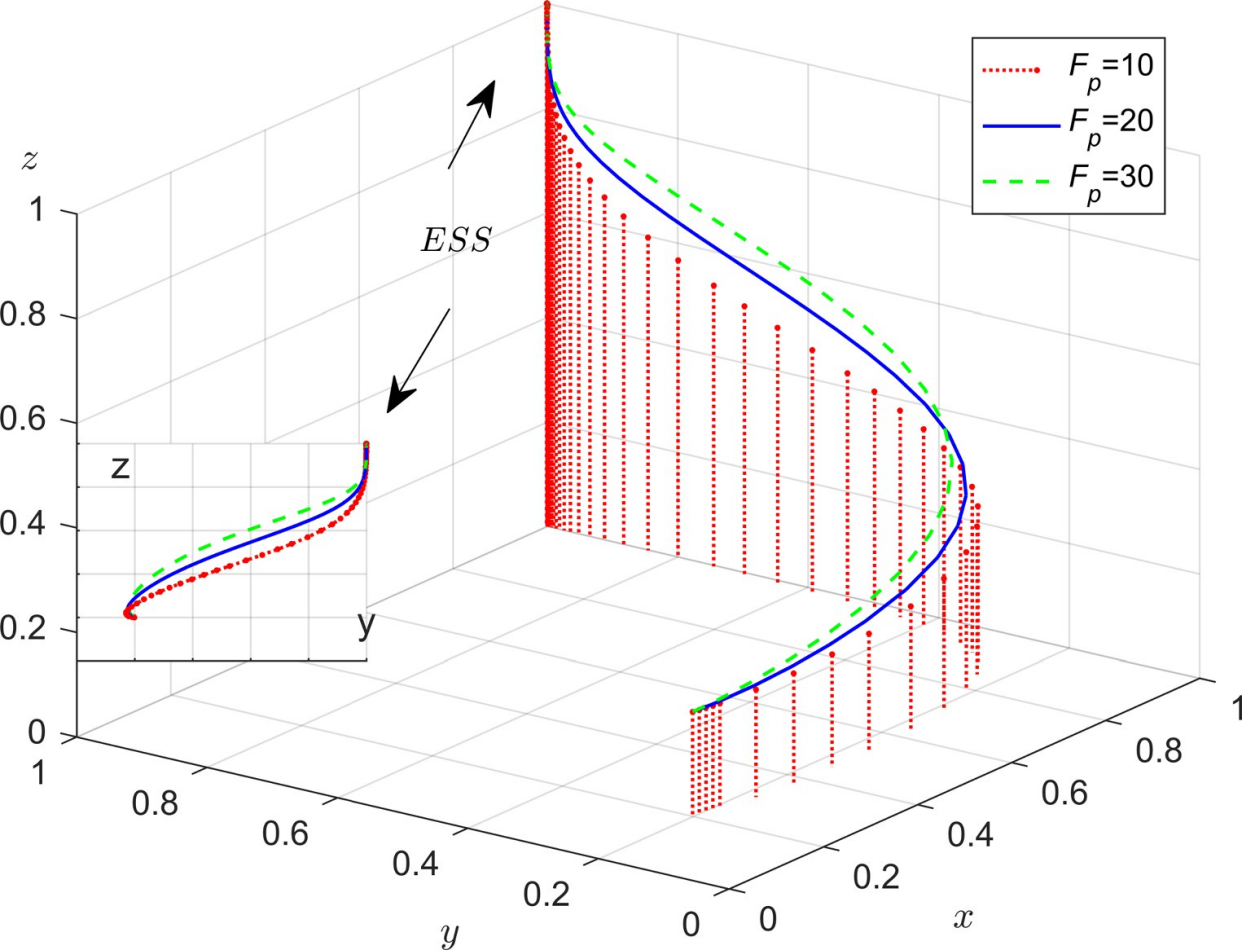
The impact of compensation to the public for pollution caused by enterprises.

From Fig 7, as the amount of government punishment for enterprises increases, it can boost the evolution speed of energy-efficient production of enterprises, leading to an increase in the probability of production enterprises adopting energy-efficient production strategies. On the other hand, when the probability of enterprises choosing energy-efficient production strategies is higher, the local governments are more inclined to choose the strategy of loose regulation; As can be seen from Fig 8, when the probability of strategic choices for enterprises and the public remains constant, with an increase in the strength of central government policies support and administrative accountability for local governments, the probability of strict supervision by local governments increases. This demonstrates that the incentive and punishment measures implemented by the central government play a crucial role in promoting the participation of local governments in regulatory governance; Fig 9 reveals that under strict government regulation, when the governance costs that the enterprises choose not to adopt energy-efficient production methods are higher, the enterprises are more inclined to choose energy-efficient production strategies. Therefore, by strengthening regulation, the local government can effectively promote energy-efficient production of enterprises; As can be seen from Fig 10, in the process of evolution, the probability of enterprises choosing energy-efficient production is more influenced by the government’s strategy choice than the public’s strategy choice. Additionally, when the probability of the governments adopting strict supervision reaches a certain level, the higher the amount enterprises pay as compensation to the public, the faster enterprises evolve towards energy saving and emission reduction.

### 4.3. The analysis of subsidies

To analyze the sensitivity of the subsidies, the parameter values in Table 7 were substituted into this model to obtain the evolutionary simulation results. And the method is similar to 4.1.

**Table 7:** The set of values of parameters in the numerical simulation of subsidies.

Parameters	*M*_*t*_	*A*_*s*_	*M*_*p*_
**Initial Values**	30	20	30
**Changed Values**	10	10	10
	50	30	50

**Fig 11:**
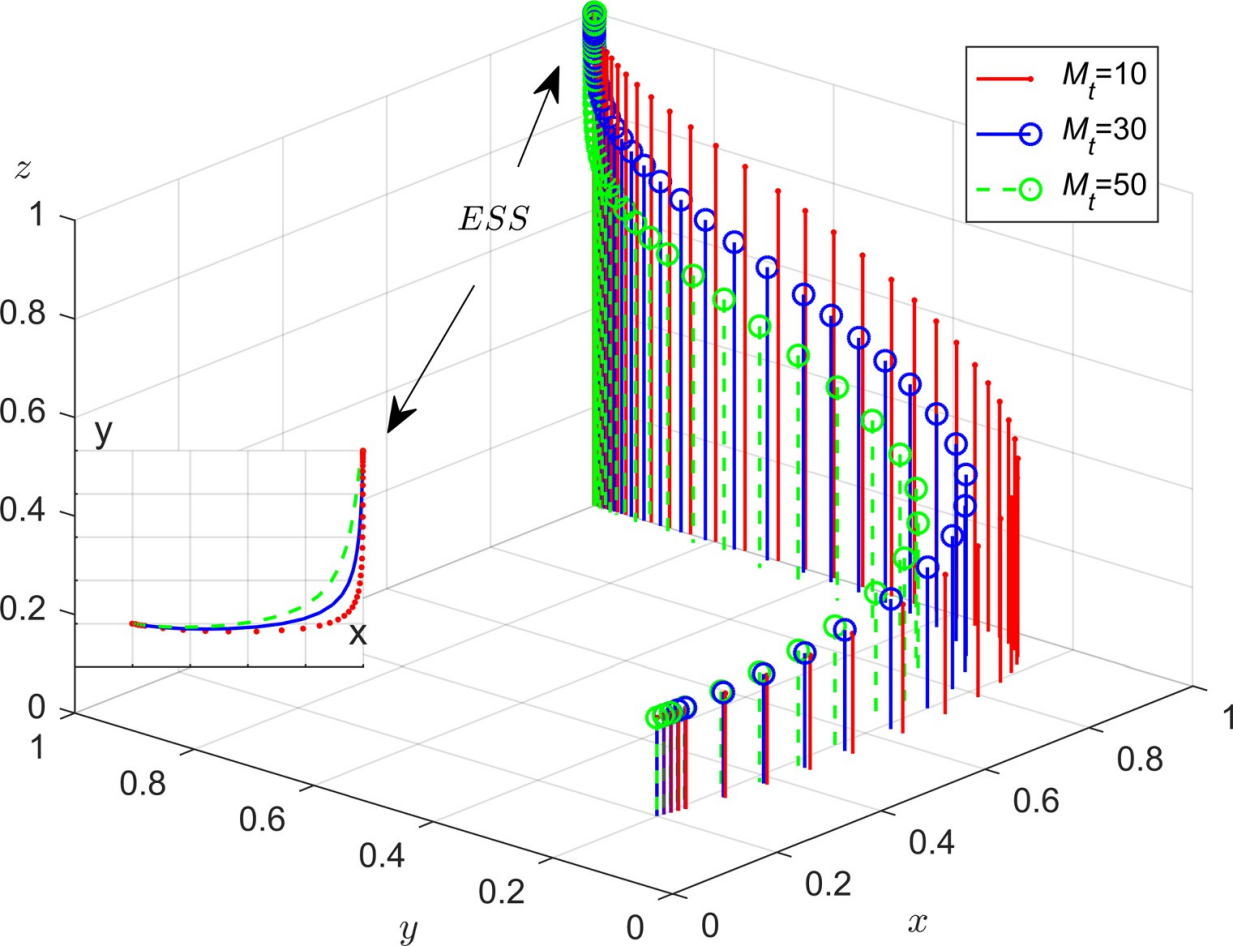
The impact of government incentives for enterprises.

**Fig 12:**
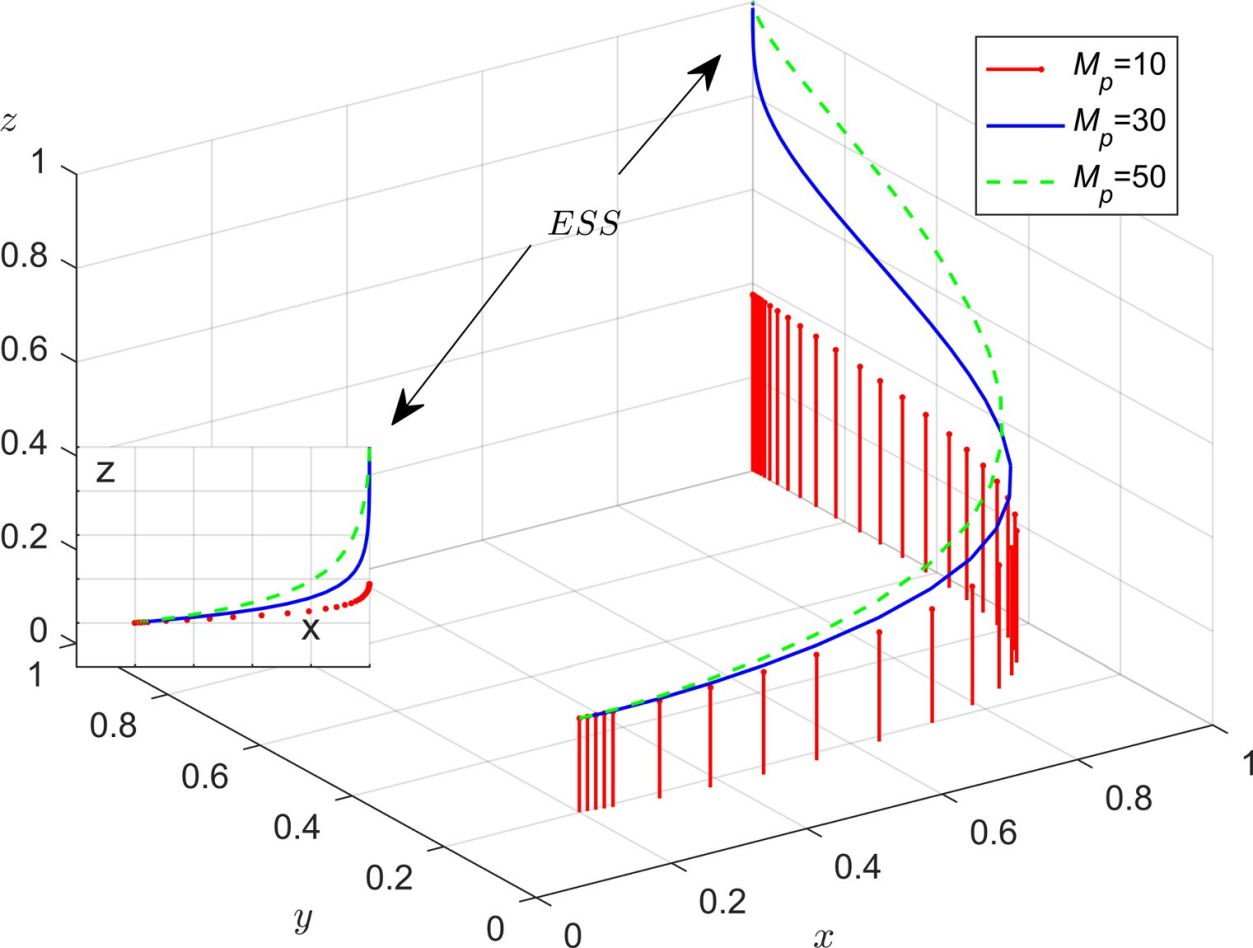
The impact that public monitoring receives incentives from the government.

As shown in Fig 11, with the increase in government rewards, the evolutionary speed of enterprises and the public participating in energy saving and emission reduction is enhanced, leading to an increase in the probability of enterprises adopting energy saving and emission reduction strategies. From Fig 12, it is evident that as the government increases the amount of the incentives for public, the probability of the public adopting a positive participation in supervision increases, while the probability of strict supervision by the government decreases. This indicates that when the public cooperates with local governments in supervision, it is advantageous for the efficient allocation of government administrative resources and enhances government management efficiency. However, when the value of *M*_*p*_ is lower, the willingness of the public to participate in the energy saving and emission reduction governance is extremely weak.

**Fig 13:**
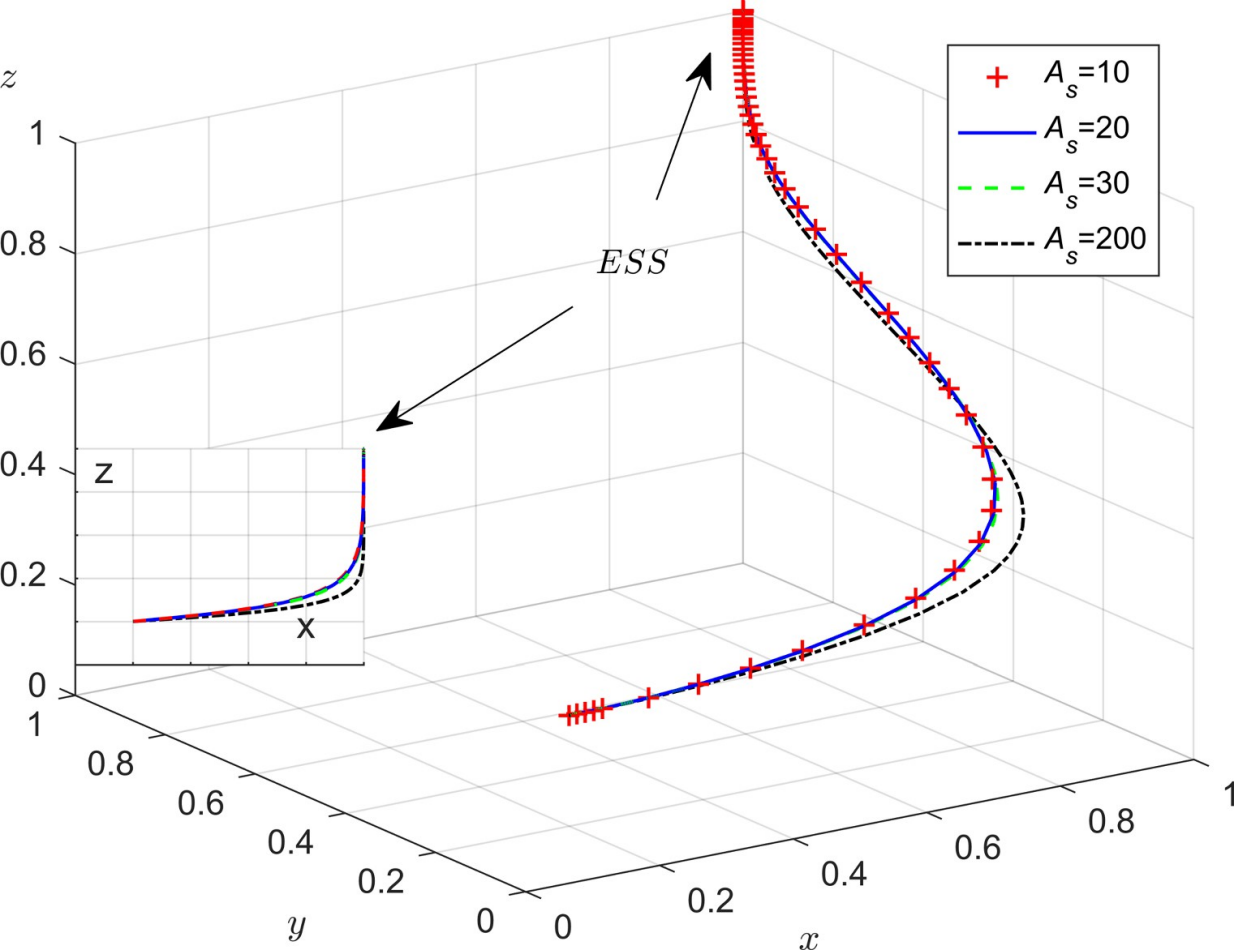
The impact of government incentives on public participation in supervision.

Fig 13 indicates that the effect of the public cooperating with the local government resulting in enhancing the government’s image benefits is not very significant. It only has a small impact on the government’s strategy selection when the public cooperation in governance results in substantial benefits for the government.

### 4.4. Impact of subsidies and penalties mechanisms on tripartite evolution trend

To further illustrate the impact of parameters on system stability in the model, numerical simulations would be used to analyze how changes in subsidies and penalties affect system stability. The values of each parameter are listed in Table 8. And the method is similar to 4.1.

**Table 8:** The set of values of parameters in the numerical simulation of subsidies and penalties.

Parameters	*M*_*t*_	*M*_*p*_	*F*_*e*_	*F*_*p*_	*A*
**Initial Values**	30	30	30	20	20
**Changed Values**	45	45	45	30	10

**Fig 14:**
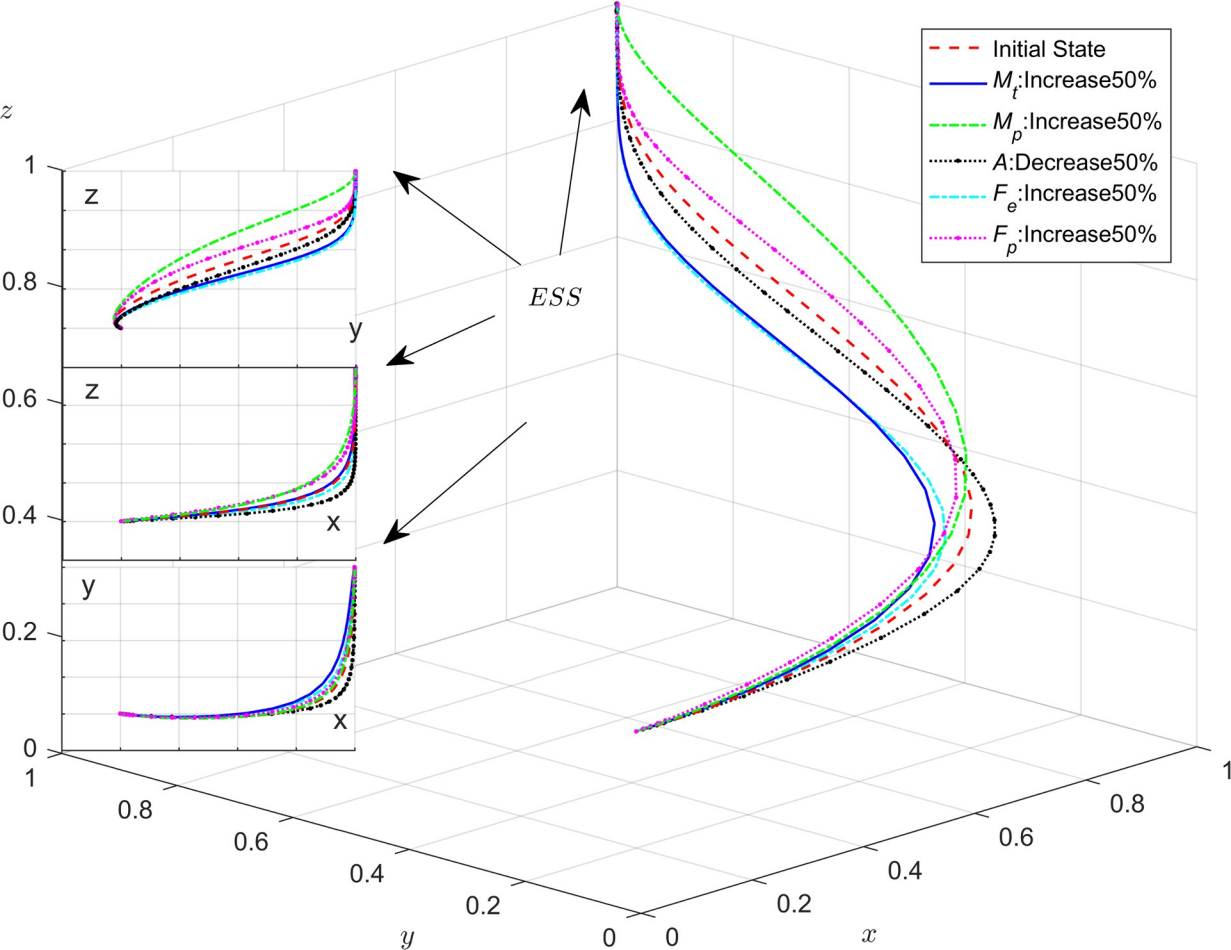
Impact of incentives and punishment mechanisms on tripartite evolution trend.

The results of these changes are presented in Fig 14. Firstly, when the system evolves to a stable point, the evolutionary trajectories of the strategic choices of enterprises and the public are more influenced by government incentives and penalties. Specifically, as the subsidies and penalties of local governments increase, the probability of enterprises choosing energy-efficient production strategies as well as the probability of the public participating in supervision both increases. However, the public is more sensitive to subsidies and the enterprises are more sensitive to penalties; Secondly, as local government subsidies and penalties for the enterprises and the public increase, the probability of the enterprises choosing energy saving and emission reduction and the public participating in monitoring will increase. In this scenario, the local government will be more inclined to choose the strategy of loose regulation; Finally, when administrative accountability and policies support from the central government is intense, local governments evolve towards strict regulatory strategies at a faster rate.

## 5. Conclusions

In the context of low-carbon economic development, advancing carbon reduction and achieving the goal of carbon neutrality has become an important issue for current and future development, requiring the active participation of multiple stakeholders. Based on exploring the stability of the equilibrium strategy of the evolutionary game, this research conducted an evolutionary analysis from the individual perspective and the holistic perspective respectively, examined the impact of the behaviors of the key stakeholders on energy conservation and emission reduction, and analyzed the impact of the changes in these key factors on the behavioral choices of each subject. The main findings are as follows: (1) In terms of the regulatory cost of energy saving and emission reduction, reducing the costs that paid by enterprises for adopting energy-efficient production can accelerate the speed of enterprises in adopting energy saving and emission reduction production policies. While for the government, the promotion of energy saving and emission reduction production policies is imperative, and the impact of the management cost is negligible. (2) Incentive and punishment mechanisms set by local governments play an important role in enhancing the motivation of enterprises and the public to participate in collaborative governance. Within the subsidy and penalty mechanism, the public demonstrated a higher sensitivity to rewards, while enterprises exhibited greater responsiveness to fines. Thus, the government should formulate a stricter penalty mechanism based on proper incentives. Increasing the penalties for not adopting energy-efficient production not only encourages the enterprises to adopt energy saving and emission reduction but also be advantageous to the local governments in the rational allocation of administrative resources. (3) Financial subsidies and administrative accountabilities from the central government are the main incentives for the local governments to strictly regulate. As the financial subsidies and administrative accountabilities of the central government to the local government become higher, the opportunity cost for the local governments to choose the loose supervision strategy becomes higher.

Based on the above research findings, this paper makes the following recommendations:

1. The central government should incorporate energy-saving and emission reduction efforts into the performance assessment system for local governments. And establish a bidirectional government assessment and evaluation system, encompassing both "top-down" and "bottom-up" evaluations. In this way, local governments can be incentivized to actively guide the green transformation of enterprises. At the same time, the local government can also provide material subsidies to the public that demonstrate active cooperation. The driving force of incentive and punishment mechanisms will enhance the willingness of both the enterprises and the public to participate, thereby maximizing the benefits of energy-saving and emission.
2. Local governments should increase subsidies and support for green transformation of enterprises, and increase penalties for enterprises that do not make green transformation, so as to promote active green transformation of enterprises. It not only facilitates the public in obtaining both social and green environmental benefits but also facilitate the government’s cross-sectoral coordination and allocation of administrative resources according to the needs of the society and the prioritization of the problems.
3. The local government can establish an open and transparent communication mechanism among all players, which not only ensures that the transmission and exchange of information is transparent and timely, but also helps to improve the mutual understanding and trust among all players. It can effectively avoid falling into the "prisoner’s dilemma" during the collaborative governance of energy conservation and emission reduction. For instance, local governments can organize public hearings, roundtable discussions, surveys, and other forms of engagement to invite businesses and the public to participate and express their opinions. Additionally, dedicated feedback channels such as hotlines, emails, or online platforms can be established to allow the public to raise questions and provide suggestions.

## 6. Limitation

This study still has some limitations. Firstly, this study is primarily based on the perspective of evolutionary game theory to explore the interactive optimization paths and stable strategies of multi-subject cooperation and win-win under energy saving and emission reduction policies. While it can validate the core position of the government, enterprises, and the public in the overall system, as well as the regulatory role of government reward and punishment mechanisms, the factors affecting carbon emissions are numerous, involving aspects such as energy efficiency, industrial structure, policy measures, and scientific and technological aspects, which also merit further discussion. secondly, in order to facilitate a more rigorous mathematical analysis, the assumptions of this model have been simplified based on the specific application domain and policies context, which may result in a limited realistic adaptation of the model. Finally, during the numerical simulation phase of the tripartite evolutionary game model, the assignment of parameters was based on equilibrium conditions, which to some extent, involves a degree of subjectivity. In future research, we will try to obtain more objective data through questionnaires or field measurements to improve the accuracy of the study and enhance the credibility of the model.

## Supporting information

S1 Program Matlab program for obtaining the eigenvalues of the 16 equilibrium. (M)

S2 Program Matlab program for plotting Fig 5. (M)
